# Breaking the Circuit: A Case of Macro–re-entrant Biatrial Tachycardia

**DOI:** 10.19102/icrm.2025.16082

**Published:** 2025-08-15

**Authors:** Mustafa Gabarin, Javier Bonacina, Syamkumar Divakara Menon

**Affiliations:** 1 Cardiology Division, Hamilton Health Sciences, Arrhythmia Service Unit, McMaster University, Hamilton, ON, Canada; 2 Cardiology Department, Meir Medical Center, Tel Aviv University, Tel Aviv, Israel

**Keywords:** Atrial fibrillation, atypical atrial flutter, catheter ablation, macro–re-entrant biatrial tachycardia

## Abstract

We present a case of a 71-year-old woman with symptomatic paroxysmal atrial fibrillation and atypical atrial flutter (AFL), ultimately diagnosed with a rare type 3 macro–re-entrant biatrial tachycardia (BiAT). Despite initial pulmonary vein isolation and anterior line ablation for atypical AFL, she experienced recurrent AFL requiring a complex redo ablation. Successful termination of the tachycardia was achieved by extending ablation to the septal regions of both atria. This case highlights the complexity of managing BiAT.

## Case presentation

**Figure 1:**
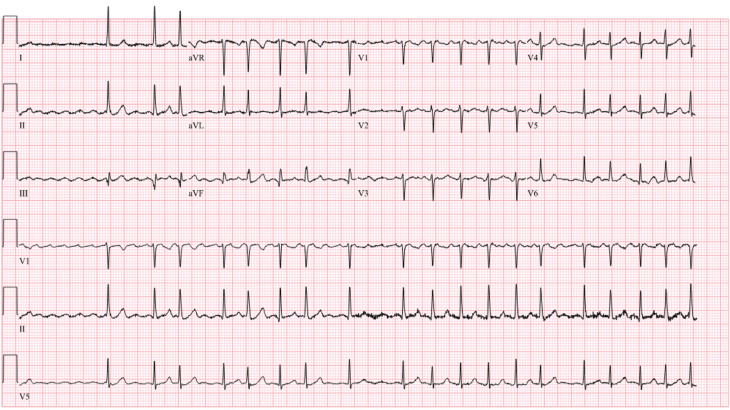
Atrial fibrillation with coarse waves.

A 71-year-old woman with systemic arterial hypertension and hypothyroidism, both well managed, was on metoprolol, flecainide, and rivaroxaban (CHA_2_DS_2_-VASc score of 3 points). She presented in February 2023 with symptomatic paroxysmal atrial fibrillation (AF). During her initial visit, she was found to be in AF **(Figure 1)**. Transthoracic echocardiography revealed normal left and right ventricular structure and function, with a left ventricular ejection fraction of 62% and a left atrial (LA) volume index of 35 mL/m^2^.

**Figure 2:**
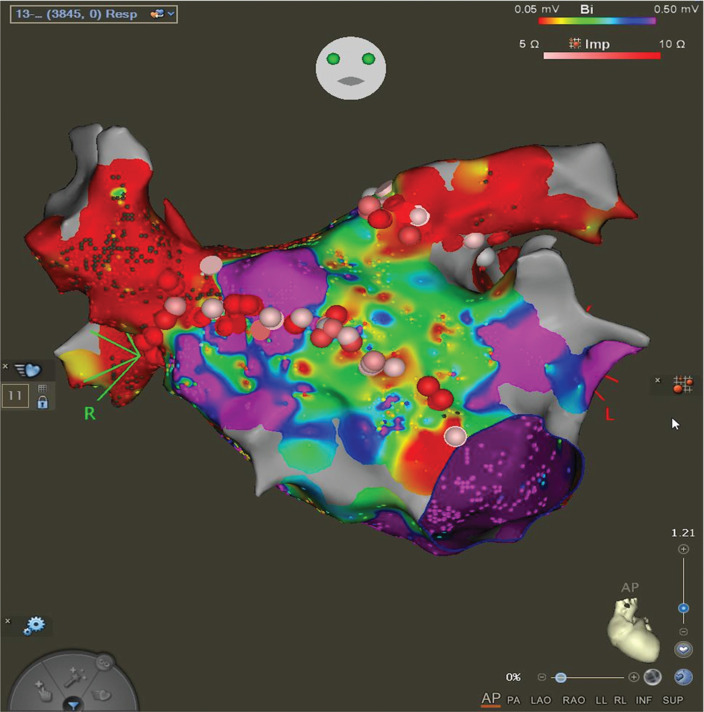
Anterior view of the left atrial voltage map after pulmonary vein isolation and anterior mitral annulus line. The first ablation procedure used a bipolar voltage map (0.05–0.5 mV) generated with a PentaRay^®^ mapping catheter (Biosense Webster). Complete pulmonary vein isolation was confirmed. An anterior line was created to target atypical atrial flutter, which was associated with scar in the anterior wall of the left atrium.

In April 2023, she underwent her first AF ablation. A bipolar voltage map revealed scarred and low-voltage areas on both the anterior and posterior walls of the LA. All pulmonary veins were isolated on the first pass. However, she subsequently developed an atrial flutter (AFL) with a tachycardia cycle length (TCL) of 250 ms. Activation mapping and entrainment confirmed a re-entrant circuit around the anterior wall scar. To address this atypical AFL, an anterior line ablation was performed from the right superior pulmonary vein, transecting the anterior wall scar to the anterior mitral annulus **(Figure 2)**. Bidirectional block was confirmed by pacing from the LA appendage and the LA septal region, showing equal conduction delay (~90 ms), suggesting conduction delay or possible block. Importantly, the atypical AFL terminated during the anterior line ablation and was not inducible thereafter. All voltage maps were created using a range of 0.05–0.5 mV.

**Figure 3:**
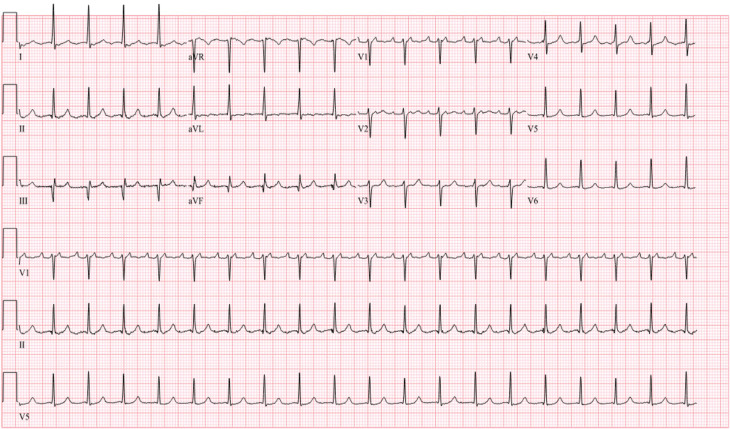
Atypical atrial flutter after first ablation; atrioventricular 2:1 conduction, 115 bpm.

Six weeks after the procedure, metoprolol and flecainide were discontinued. However, during the blanking period, the patient experienced recurrent symptomatic atypical AFL **(Figure 3)**. Despite maximal tolerated doses of β-blockers and digoxin for rate control, she remained in AFL with a heart rate of approximately 110 bpm. She was also intolerant to anti-arrhythmic drugs, including sotalol and amiodarone. Due to persistent symptoms, a redo ablation was scheduled for June 2024.

In June 2024, the patient underwent a redo ablation for atrial tachycardia (AT). The OCTARAY™ high-density mapping catheter (Biosense Webster, Diamond Bar, CA, USA), THERMOCOOL SMARTTOUCH™ SF ablation catheter (Biosense Webster), and intracardiac echocardiography were used under the guidance of a three-dimensional electroanatomic mapping system (CARTO™; Biosense Webster). The contact force during ablation ranged from 10–25 g on the anterior septal LA and from 5–15 g on the posterior septal right atrium (RA). The power was set at 50 W. The impedance drops across ablation points ranged from 10–18 Ω.

**Figure 4:**
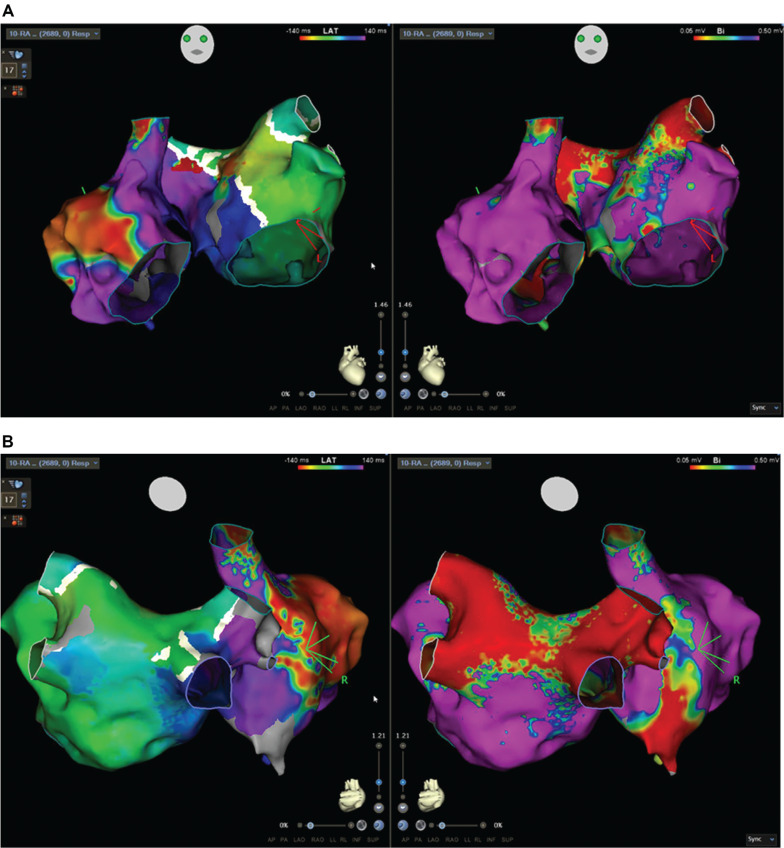
Bipolar voltage and activation mapping of both atria during atrial tachycardia in the **(A)** anterior view and **(B)** posterior view. The left panels show activation maps encompassing the entire tachycardia cycle length (280 ms) across both atria. The right panels show bipolar voltage maps with settings of 0.05–0.5 mV, demonstrating normal and low-voltage areas.

All four pulmonary veins and the posterior LA wall were confirmed to be isolated, and the prior anterior LA ablation line showed bidirectional conduction delay. A bipolar voltage map confirmed isolation of the pulmonary veins and posterior LA, with additional scarring and low-voltage areas on the anterior LA wall. Activation mapping revealed that 70% of the TCL was localized to the LA. However, further mapping of the RA was performed, as the entire TCL could not be accounted for in the LA alone. Bipolar voltage mapping of the RA revealed low-voltage areas and scarring in the posterior septal region extending toward the inferior vena cava. When analyzing the activation map from the anterior view, the earliest activation appeared to originate from the anterolateral RA. This highlights an important pitfall to consider carefully—what occurred in this case was a late activation that falsely appeared early within the window of interest on the activation map. However, entrainment mapping ruled out this site as the true earliest activation area **(Figures 4A and 4B)**.

Entrainment mapping identified a macro–re-entrant AFL with a TCL of 280 ms. The earliest activation on the coronary sinus (CS) catheter was noted distally (CS 1, 2). Entrainment from both proximal and distal CS sites indicated that neither site was part of the re-entry circuit (post-pacing interval [PPI] to TCL difference > 30 ms). Further activation mapping revealed that most of the TCL was recorded in the LA.

**Figure 5:**
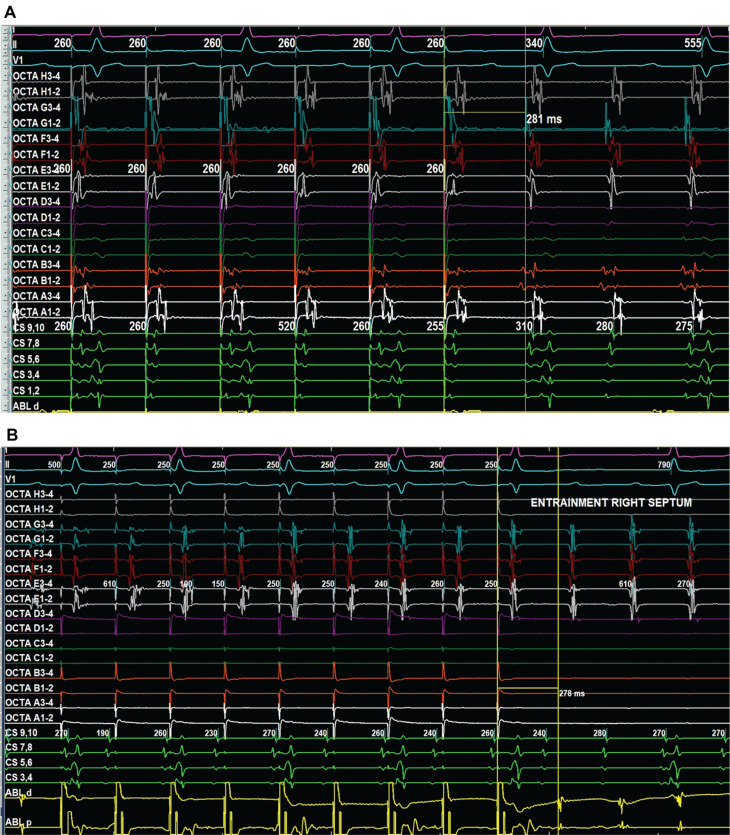
**A:** Septal left atrial entrainment by OCTARAY™ mapping catheter electrode E3–4 showed post-pacing interval (280 ms) – tachycardia cycle length (280 ms) = 0 ms (sweep speed, 150 mm/s). **B:** Septal right atrial entrainment by ablation catheter showed a post-pacing interval−tachycardia cycle length of 0 ms (sweep speed, 100 mm/s).

Additional entrainment mapping in the LA septal region and posterior RA septum confirmed a PPI–TCL of 0 ms **(Figures 5A and 5B)**, with adjacent areas showing a PPI–TCL difference of <30 ms, confirming these regions as part of the re-entrant circuit. Initially, fragmented low-voltage signals near the anterior LA wall, adjacent to the prior ablation line, raised suspicion of involvement based on propagation mapping. However, entrainment mapping at this site demonstrated a PPI–TCL difference of >30 ms, and ablation here did not terminate the flutter.

**Figure 6:**
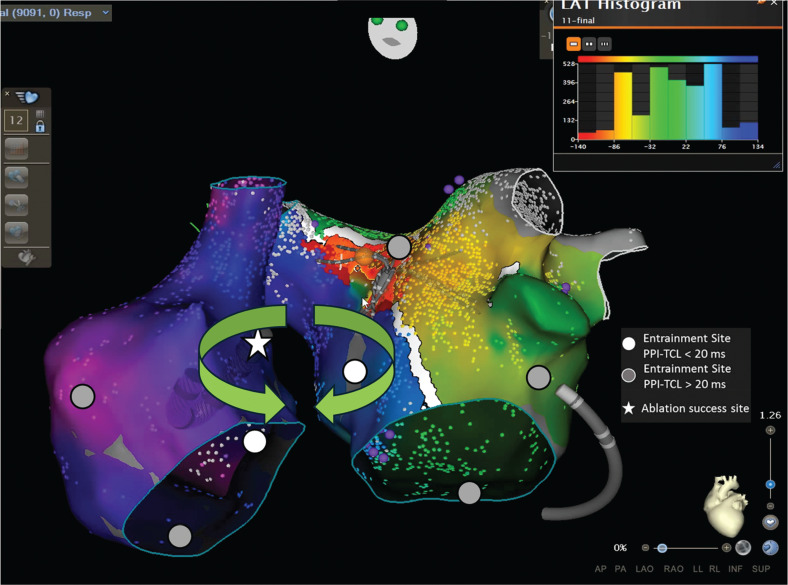
Activation map during tachycardia (cycle length, 280 ms) of both atria. The activation map demonstrates complete tachycardia cycle mapping in both atria, with the earliest activation near the anterior right upper pulmonary vein, likely due to prior anterior mitral isthmus line ablation. Entrainment mapping confirmed out-of-circuit sites (gray dots) and circuit sites (white dots). The circuit involved the septa of both atria (green arrows). Successful ablation was achieved at the right posterior septal right atrium (star marker).

**Figure 7:**
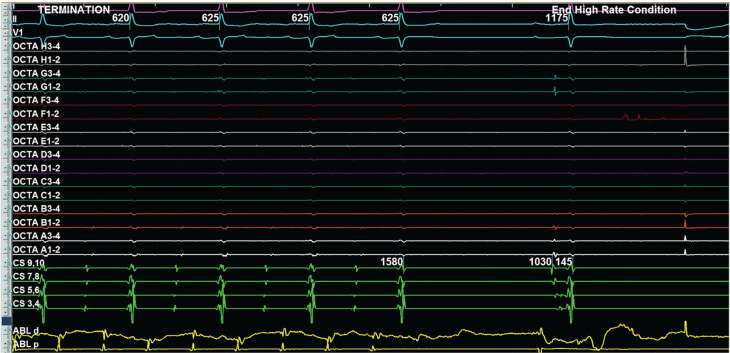
Tachycardia termination during catheter ablation on the septal right atrium (sweep speed, 150 mm/s).

Propagation mapping further confirmed a macro–re-entrant biatrial flutter involving the septal regions of both atria **(Video 1, Figure 6)**. Initial ablation targeted the LA septal regions but failed to terminate the flutter. Ablation was then extended to the RA septal regions, parallel to the left-sided septal ablation, which successfully terminated the tachycardia **(Video 2, Figure 7)**. Post-ablation, complex fractionated atrial electrograms were detected, and AT was no longer inducible.

## Discussion

Biatrial tachycardia (BiAT) represents a rare subset of atypical AT, accounting for 0.5%–2.1% of cases in these categories characterized by a re-entrant circuit that spans both atria, frequently observed in patients with a history of prior cardiac surgeries or AF and AFL ablation.^1,2^ Kitamura et al. described three types of biatrial flutter circuits; our case aligns with type III, where both the right and left septa are active parts of the circuit, while remaining parts of the atria are passively activated as bystanders.^2^ The interatrial electrical connection of the RA and LA relies on both endocardial and epicardial insertions, involving structures such as Bachmann’s bundle, the septopulmonary bundle, the CS, and anterosuperior and posteroinferior interatrial septal connections.^3,4^ In their study, nine BiATs were identified in eight patients (2.1% of mapped ATs), mostly with prior AF ablation. Termination was achieved in 88.9% by targeting specific isthmuses or interatrial connections, such as the cavotricuspid isthmus, septal scars, or high RA septum. These findings support tailored ablation strategies for BiAT.

High-resolution local activation and bipolar voltage maps, along with targeted entrainment, are crucial for identifying the re-entrant pathways in atypical AFL, which may include an inner loop, isthmus, outer loop, and bystander regions. In our case, electroanatomic mapping revealed unhealthy areas and scarring in the anterior LA from prior procedures, with bidirectional conduction delay at the anterior mitral isthmus line. Active mapping during BiAT in both atria demonstrated that most of the TCL originated in the LA, and propagation maps showed a deceleration zone acting as an isthmus in the anterosuperior LA near the right superior pulmonary vein. Entrainment mapping was essential for identifying the circuit; however, contrary to expectations, entrainment in the presumed area of interest was outside the circuit. High-fractionated, low-amplitude signals were identified at the septum of both the LA and RA. Entrainment in the LA septum was part of the tachycardia circuit, and entrainment at the corresponding RA posterior septal site demonstrated a PPI–TCL of 0 ms, confirming circuit involvement. Ablation at both atrial septal sites terminated the tachycardia without reinduction, substantiating the diagnosis of type III BiAT **(Figure 6)**.

This case highlights the significance of recognizing biatrial flutter circuits and tailoring ablation strategies accordingly. High-density mapping and entrainment are invaluable in pinpointing slow-conducting regions that function as critical isthmuses in macro–re-entrant circuits, thereby improving ablation outcomes in complex AFL cases.

## Conclusion

This case underscores the complexity of managing macro–re-entrant biatrial flutter, particularly when previous ablation attempts succeeded. Extending ablation to septal regions in both atria can be effective in terminating type 3 BiAT, emphasizing that activation mapping of both atria is essential, with entrainment playing a key role in identifying the circuit. Integrating all available data is crucial for achieving an accurate diagnosis.

## Supporting information

Video 1: Atrial tachycardia propagation map for both atria.

Video 2: Tachycardia termination during catheter ablation on septum of right atrium.
